# Variability between Clarke's angle and Chippaux-Smirak index for the diagnosis of flat feet

**Published:** 2017-03-30

**Authors:** Cristina Gonzalez-Martin, Salvador Pita-Fernandez, Teresa Seoane-Pillado, Beatriz Lopez-Calviño, Sonia Pertega-Diaz, Vicente Gil-Guillen

**Affiliations:** 1 Clinical Epidemiology Research Group, Health Sciences Department, Escuela Universitaria de Enfermería y Podología, Universidade da Coruña (UDC), Ferrol, Spain; 2 Clinical Epidemiology and Biostatistics Research Group, Instituto de Investigación Biomédica de A Coruña (INIBIC), Complexo Hospitalario Universitario de A Coruña (CHUAC), SERGAS, Universidade da Coruña, Coruña, Spain; 3 Department of Clinical Medicine, Universidad Miguel Hernandez, Alicante, Spain

**Keywords:** Footprint, Pedigraph, Chippaux-Smirak index, Clarke's angle, Flatfoot, Foot, Sensitivity and Specificity, Anthropometry, Podiatry

## Abstract

**Background:**

The measurements used in diagnosing biomechanical pathologies vary greatly. The aim of this study was to determine the concordance between Clarke's angle and Chippaux-Smirak index, and to determine the validity of Clarke's angle using the Chippaux-Smirak index as a reference.

**Methods:**

Observational study in a random population sample (n= 1,002) in A Coruña (Spain). After informed patient consent and ethical review approval, a study was conducted of anthropometric variables, Charlson comorbidity score, and podiatric examination (Clarke's angle and Chippaux-Smirak index). Descriptive analysis and multivariate logistic regression were performed.

**Results:**

The prevalence of flat feet, using a podoscope, was 19.0% for the left foot and 18.9% for the right foot, increasing with age. The prevalence of flat feet according to the Chippaux-Smirak index or Clarke's angle increases significantly, reaching 62.0% and 29.7% respectively. The concordance (kappa I) between the indices according to age groups varied between 0.25-0.33 (left foot) and 0.21-0.30 (right foot). The intraclass correlation coefficient (ICC) between the Chippaux-Smirak index and Clarke's angle was -0.445 (left foot) and -0.424 (right foot). After adjusting for age, body mass index (BMI), comorbidity score and gender, the only variable with an independent effect to predict discordance was the BMI (OR= 0.969; 95% CI: 0.940-0.998).

**Conclusion:**

There is little concordance between the indices studied for the purpose of diagnosing foot arch pathologies. In turn, Clarke's angle has a limited sensitivity in diagnosing flat feet, using the Chippaux-Smirak index as a reference. This discordance decreases with higher BMI values.

## Resumen

**Introducción:**

Existe una gran variabilidad en las mediciones para el diagnóstico de la patología biomecánica. El objetivo de este estudio fue determinar la concordancia entre el ángulo de Clarke y el índice de Chippaux-Smirak, para determinar la validez del ángulo de Clarke utilizando como referencia el índice de Chippaux-Smirak.

**Métodos:**

Se realizó un estudio observacional en una muestra aleatoriamente seleccionada (n=1,002) en A Coruña (España). Tras el consentimiento informado del paciente y la aprobación del comité de ética, se estudiaron variables, antropométricas, índice de comorbilidad de Charlson y un examen podológico (ángulo de Clarke, índice de Chippaux-Smirak). Se realizó un estudio descriptivo y un análisis multivariado de regresión logística.

**Resultados:**

La prevalencia de pie plano utilizando el podoscopio fue de 19.0% (pie izquierdo) y 18.9% (pie derecho), incrementándose con la edad.La prevalencia de pie plano según el índice Chippaux-Smirak o el ángulo de Clarke se incrementan considerablemente llegando a 62.0% y 29.7%.La concordancia (kappa I) entre los índices según grupos de edad oscila entre 0.25-0.33 (pie izquierdo) y 0.21-0.30 (pie derecho). El coeficiente de correlación intraclase (CCI) entre el índice de Chippaux-Smirak y el ángulo de Clarke es -0.445 (pie izquierdo) y 0.424 (pie derecho). Tras ajustar por edad, índice de masa corporal (IMC), score de comorbilidad y sexo la única variable con un efecto independiente para predecir discordancia es el IMC (OR= 0.969; IC 95%: 0.940-0.998).

**Conclusiones:**

La concordancia entre los índices estudiados para el diagnóstico de la patología del arco plantar es reducida. Existe a su vez una reducida sensibilidad del ángulo de Clarke para el diagnóstico de pie plano, utilizando como referencia el índice de Chippaux-Smirak. Esta discordancia disminuye con valores más altos de IMC.

## Introduction

Clinical practice is not only affected by variability and uncertainty in the process of taking therapeutic and prognostic decisions, but also when taking decisions with regard to diagnosing the presence or absence of a given pathology ^1^. In clinical practice, variability may be present in practically every stage of the process, equally affecting the field of podology, as there are diagnostic tests that are modified by the variability of the observers and the different tests that confirm the presence or absence of the pathology. With regard to flat feet, the first hurdle is that there is no universally accepted definition for pes planus. Clinically, a pes planus is a foot that has a low or absent longitudinal arch ^2^. 

Historically, a series of parameters have been used to study the pathology and morphology of the foot, such as angles, indices and lines obtained from the footprint. Schwartz was the first researcher to create a series of angles based on the footprint in order to determine if a foot was normal or suffered from some type of pathology ^3^. Since then, a wide range of methods have been used with the aim of determining the morphology of the foot and diagnosing foot pathologies.

A valid, simple way of analysing the footprint is by using a pedigraph ^4^. Podoscopes and pedigraphs are normally used in order to study the prevalence of biomechanical alterations.

Different procedures are used to diagnose flat feet: physical examinations (mainly of the medial longitudinal arch and heel angle), photography, footprints, gait analysis/plantar pressures and pedobarograph measurements ^2^. In turn, different scales are used to study the footprint, such as the valgus index, arch index, Staheli arch index, visual assessment, Chippaux-Smirak index, Foot posture index and Clarke's angle ^5^, which increase the variability. In general, the reliability of these measurements is poor. 

Some authors recommend using the Chippaux-Smirak index as a screening instrument for flat feet in preschool-aged children ^6^, although there is no sound support for either continuing or establishing podiatry screenings for children ^7^. The Chippaux-Smirak index has been used as the standard assessment tool for determining whether preschool-aged children suffered from flat feet ^8^. The validity of the most commonly used foot print analysis methods for diagnosing flatfoot, using clinical diagnosis as a gold standard was published for our group previously ^9^.

We carried out this study with the aim of determining the concordance between Clark's angle and the Chippaux-Smirak index, and determining the validity of Clarke's angle using the Chippaux-Smirak index as a reference for the diagnosis of flat feet.

## Materials and Methods

### Setting and study population

A cross-sectional study was conducted between November 2009 and July 2012 on a random population sample from Cambre (A Coruña-Spain) (Local Council of Cambre; http://www.cambre.org/). 

### Sampling, recruitment and inclusion criteria

The sampling frame consisted of individuals resident in Cambre identified through the National Health System card census. In Spain, the National Health System has universal coverage and almost all Spanish citizens are beneficiaries of public health services. The inclusion criteria were being 40 years of age or older, and having provided informed consent. The sample was randomly selected, once stratified by age and gender. The participants were sent a personal letter explaining the purpose of the study and the examinations that would be carried out. They were then contacted by telephone to arrange an appointment at the health centre. 

### Sample size justification

The sample size was calculated taking into account the total population of the municipality (n= 23,649) after stratification by age and gender. Finally 1,002 persons were included in the study. This sample size (n= 1,002 persons; 505 between the ages of 40 and 64, and 497 who were 65 and older) makes it possible to estimate the parameters of interest with a confidence of 95% (α = 0.05) and a precision of ± 5%, assuming an information loss of 15%.

### Measurements

The following variables were studied: anthropometric variables (age, gender and body mass index), study of chronic comorbidities with the Charlson comorbidity index and podiatric examination.

The Charlson Index contains 19 categories of comorbidity, which are primarily defined using ICD-9-CM diagnosis codes. Each category has an associated weight, taken from the original Charlson paper ^10^, based on the adjusted risk of one-year mortality. The overall comorbidity score reflects the cumulative increased likelihood of one-year mortality; the higher the score, the more severe the burden of comorbidity.

The podiatric examination, performed by an experienced podologist, included:

Study of the footprint obtained with a pedigraph. The footprints were obtained by placing a reticulated piece of rubber sheeting, tensed and impregnated with ink, between the subject's foot and a piece of stretched paper. In order to obtain the footprint, a footprint ink mat was used (podograph). To study the footprint by pedigraph, two footprint measurements were used: Clarke's angle, and the Chippaux-Smirak index ^11^.

The validity of these three foot print measurements in comparison with clinical diagnoses has been described previously ^12^.

The reliability and validity of the measurements used in this study have been described by different authors ^13^ ^,^ ^14^. In a literature review of the reliability and validity of the current physical examination ^13^, a wide variability was identified depending of the examination performed. The measurements used in this case are the ones that are recommended to perform a clinical examination of the foot and ankle. 

The measurements taken on the imprint were Clarke's angle and Chippaux-Smirak index ^15^. 

A study of the arches, foot shape, metatarsal shape, signs in shoe-wear patterns, forefoot to rearfoot relationship, rearfoot position and foot deformities will be the subject of further research.

### Statistical analysis

The quantitative variables are expressed as a mean (Standard Deviation); the qualitative variables are expressed as an absolute value (n) and the percentage, with the estimation of the 95% confidence interval (CI). Comparisons for quantitative variables were made using the Student-T or Mann Whitney test, depending on which was appropriate after checking for normality using the Kolgomorov-Smirnov test. Qualitative variables associations were analysed using Pearson's Chi-Square test. 

The correlation was determined using Pearson's or Spearman's Rho correlation coefficient; the agreement was determined by the intraclass correlation coefficient, and concordance using the kappa index.

Using the Chippaux-Smirak index as the benchmark for the diagnosis of flat feet, the sensitivity, specificity and predictive values of Clarke's angle were calculated for the diagnosis of these pathologies. 

Fagan's nomogram was plotted to visualise the likelihood ratio of a test with a patient's pre-test probability of disease in order to estimate post-test probability ^16^.

In order to account for different variables a logistic regression analysis was used to examine factors associated with disagreement. All statistical analyses were performed using SPSS^®^ 19.0

### Ethics

The study complies with the principles laid down in the Declaration of Helsinki. Informed consent was obtained from all the participants in the study. Confidentiality was preserved in accordance with the current Spanish Data Protection Law (15/1999). The study has received written approval from the regional Ethics Committee for Clinical Research (code 2008/264 CEIC Galicia).

## Results

The characteristics of the sample of 1,002 people that was studied are shown in Table 1, showing a mean age of 62.3 (13.1) years with a range of between 41 and 96 years. Comorbidity increases progressively and significantly with age, in the same way as the body mass index. The prevalence of obesity in the group aged 65 and over reached a value of 45.9%, significantly higher than in the younger group between 40 and 64 years of age.

**Table 1:** General characteristics of the total sample.

	Total(n= 1,002)	40-64 years (n= 505)	&‌#8805;65 years(n= 497)	
	Mean±SD	Mean±SD	Mean±SD	*p*
Age (years)	62.33±13.14	51.02±6.79	73.82±6.25	<0.001
Charlson comorbidity index	2.17± 1.79	0.86± 0.99	3.57± 1.34	<0.001
BMI (kg/m2)	29.19±4.74	28.42±4.86	29.96±4.48	<0.001
	n (%)	n (%)	n (%)	
BMI Categories				
Normal weight(18.5kg/m2≤BMI<25kg/m2)	187 (18.8)	127 (25.3)	60 (12.1)	
Overweight (25kg/m2≤BMI<30kg/m2)	416 (41.8)	208 (41.5)	208 (42.0)	
Obesity (BMI≥30kg/m2)	393 (39.5)	166 (33.1)	227 (45.9)	<0.001
Gender				
Male	471 (47.0)	236 (46.7)	235 (47.3)	
Female	531 (53.0)	269 (53.3)	262 (52.7)	0.861
Left Footprint				
Flat foot	188 (19.0)	62 (12.4)	126 (25.8)	<0.001
High arch foot	127 (12.8)	85 (17.0)	42 (8.6)	<0.001
Normal foot	675 (68.2)	354 (70.7)	321 (65.6)	<0.001
Right Footprint				
Flat foot	187 (18.9)	61 (12.2)	126 (25.8)	<0.001
High arch foot	112 (11.3)	76 (15.2)	36 (7.4)	<0.001
Normal foot	691 (69.8)	364 (72.7)	327 (66.9)	<0.001
Chippaux-Smirak index				
Flat foot (>45%)	596 (62.0)	267 (55.2)	329 (68.8)	<0.001
High arch foot (≤25%)	69 (7.2)	46 (9.5)	23 (4.8)	0.005
Normal foot (26-45%)	455 (47.3)	268 (55.4)	187 (39.1)	<0.001
Clarke´s angle				
Flat foot (≤30º)	286 (29.7)	95 (19.6)	191 (40.0)	<0.001
High arch foot (>45º)	237 (24.6)	154 (31.8)	83 (17.4)	<0.001
Normal foot (31-45º)	680 (70.7)	369 (76.2)	311 (65.1)	<0.001

BMI: Body Mass Indice

There is a slight predominance of women in sample, corresponding to the population structure by age groups.

For the footprint study, we had data for 963 people for the left foot and 962 for the right foot, as one person had suffered an amputation of the right leg. 

The prevalence of flat feet, pes cavus and normal feet is shown in Table 1. The prevalence of flat feet in the left footprint using the podoscope is 19.0% and 18.9% in the right foot, with this prevalence increasing with age.

The prevalence of flat feet and pes cavus according to the Chippaux-Smirak index and Clarke's index in the sample as a whole and by age groups is also shown in Table 1.

The prevalence of flat feet according to the Chippaux-Smirak index is 62.0%. This increases significantly with age, reaching 68.8% of the sample over the age of 64. The same occurred using Clarke's angle for the diagnosis of flat feet, with a prevalence for the whole of the sample of 29.7%, reaching 40% in the group of subjects over the age of 64. The prevalence of pes cavus using both the Chippaux-Smirak index and Clarke's angle decreases significantly with age.

The Chippaux-Smirak index detected a higher prevalence of flat feet than Clarke's angle, while in turn Clarke's angle detected a higher prevalence of pes cavus than the Chippaux-Smirak index.

Table 2 shows the concordance between the indices for the diagnosis of flat feet, pes cavus or normal feet in the sample as a whole by the foot and by age groups. This concordance for the left foot has a kappa index that varies according to age groups by between 0.25 and 0.33, with an observed concordance that varies between 53.5% and 60.3%. For the right foot, this concordance has a kappa index that varies between 0.21 and 0.30, and an observed concordance that varies between 50.8% and 57.1%.

**Table 2:** Concordance between Chippaux-Smirak index and Clarke´s angle for groups of age according to foot.

	Chippaux-Smirak index (CSI)			Kappa Index (95%CI)	Observed Concordance (%)
Clarke´s angle (CA)	High arch foot (CSI ≤25º)	Normal foot (25º ≤CSI ≤45º)	Flat foot (CSI> 45º)		
Left Foot					
40 to 64 years (n= 484)					
High arch foot (CA > 45º)	28	9	0	0.258 (0.20-0.32)	53.5
Normal foot (30º ≤CA≤45º)	49	157	67		
Flat foot (CA ≤30º)	20	141	74		
65 or higher (n= 479)					
High arch foot (CA > 45º)	11	4	1	0.329 (0.26-0.39)	60.3
Normal foot (30º ≤CA&‌45º)	26	119	11		
Flat foot (CA≤30º)	11	137	159		
Total sample (n= 963)					
High arch foot (CA > 45º)	39	13	1	0.305 (0.26-0.35)	56.9
Normal foot (30º ≤CA≤45º)	75	276	17		
Flat foot (CA≤30º)	31	278	233		
Right Foot					
40 to 64 years (n= 484)					
High arch foot (CA > 45º)	27	7	0	0.217 (0.16-0.28)	50.8
Normal foot (30º ≤CA≤45º)	57	160	11		
Flat foot (CA≤30º)	33	130	59		
65 or higher (n= 479)					
High arch foot (CA > 45º)	16	1	0	0.303 (0.24-0.37)	57.1
Normal foot (30º ≤CA≤45º)	30	116	12		
Flat foot (CA≤30º)	23	139	141		
Total sample (n= 963)					
High arch foot (CA > 45º)	43	8	0	0.272 (0.23-0.32)	53.9
Normal foot (30º ≤CA≤45º)	87	276	23		
Flat foot (CA≤30º)	56	269	200		

The intraclass correlation coefficient between the Chippaux-Smirak index and Clarke's angle was -0.445 for the left foot and -0.424 for the right foot. The intraclass correlation coefficient between Clarke's angle and the Staheli index and the Chippaux-Smirak index and Staheli index is not significant.

We used the Chippaux-Smirak index as a reference for the diagnosis of flat feet, and studied the validity of Clarke's angle in making this diagnosis. The results are shown in Table 3. 

**Table 3:** Validity of Clarke´s angle (cut-off point ≤ 30º).

	Left Foot			Right Foot		
	Criterion of reference (Chippaux-Smirak index)					
	Flat Foot (CSI>;45º)	Not flat foot (CSI&‌#8804;45º)	Total	Flat Foot (CSI>45º)	Not flat foot (CSI≤45º)	Total
Diagnostic test results (Clarke´s angle)						
Flat Foot (CA≤30º)	304	92	396	299	110	409
Not flat foot (CA>30º)	291	276	567	277	276	553
Total	595	368	963	576	386	962
	%	95% CI		%	95% CI	
		Lower Limit	Upper Limit		Lower Limit	Upper Limit
Prevalence of disease	61.79	58.62	64.85	59.88	56.69	62.98
Patients diagnosed correctly	60.23	57.05	63.32	59.77	56.59	62.88
Sensitivity	51.09	47.00	55.17	51.91	47.74	56.05
Specificity	75.00	70.19	79.28	71.50	66.67	75.90
Positive Predictive value	76.77	72.23	80.78	73.11	68.48	77.29
Negative predictive value	48.68	44.50	52.87	49.91	45.67	54.15
Negative likelihood ratio	2.04	1.68	2.48	1.82	1.53	2.17
Positive likelihood ratio	0.65	0.59	0.72	0.67	0.61	0.75
Area Under the Curve	0.693	0.661	0.726	0.656	0.622	0.690
Youden Index	0.35	0.30	0.39	0.23	0.17	0.29

In the left foot, we found that the sensitivity for the diagnosis of flat feet is 51.1% and the specificity 75.0%, with a positive predictive value of 76.8% and a negative predictive value of 48.7%. In other words, the likelihood of an individual with flat feet obtaining a positive test with Clarke's angle is 51.1% (the proportion of true positives that are correctly identified by the test - sensitivity), while the likelihood of an individual without flat feet obtaining a negative result is 75.0% (the proportion of true negatives that are correctly identified by the test -specificity); the likelihood of having flat feet on obtaining a positive result with Clarke's angle is 76.8% (positive predictive value), and the likelihood of an individual with a negative result of not really having flat feet is 48.7% (negative predictive value).

**Figure 1:**
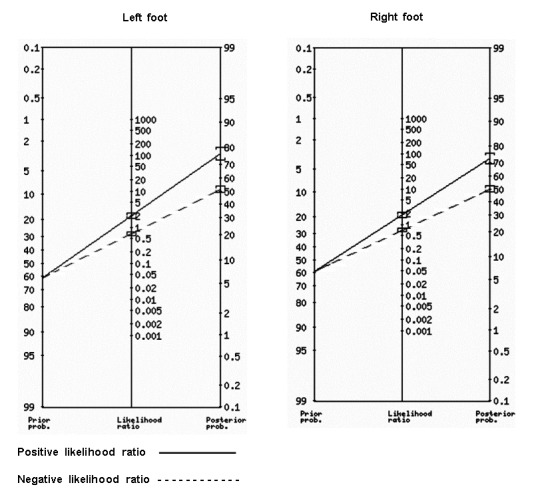
Positive and negative likelihood ratio for the diagnosis of flat feet by foot

The pre-test probability of the left foot was found to be 61.8%, the post-test probability was 76.8%, the positive likelihood ratio was 2.04 and the negative likelihood ratio was 0.65 (Fig. 1, Table 3). Similar results were obtained for the right foot.

The positive likelihood ratio is 2.04, which refers to how many times it is more likely that the test will be positive in a patient who has the disorder than one who does not, while the negative likelihood ratio is 0.65 (Fig. 1). Similar results were obtained for the right foot.

Table 4 shows the characteristics of the study group depending on whether the measurements for the diagnosis of flat feet coincided with the indices that were studied. The table shows that the patients in whom the measurements coincide are significantly older, with a higher body mass index and comorbidity score, without any significant differences in terms of gender. In order to determine whether the variables are associated with the presence of discordance, we carried out a multivariate logistical regression analysis, considering age, BMI, Charlson comorbidity score and gender. After adjusting for these variables (Table 4), we verified that the only variable with an independent effect to predict discordance is the BMI, which has a protective effect: the higher the BMI, the lower the likelihood of discordance (OR= 0.969; 95% CI: 0.940-0.998) 

**Table 4:** Patients characteristics according the concordance of diagnosis of flat foot between Chippaux-Smirak index and Clarke´s angle and adjusted odds ratio to predict discordance

Variables	Concordance YES	Concordance NO	p*	Adjusted OR**	95% CI OR
	Mean±SD	Mean±SD			
Age (years)	63.22±13.08	60.91±12.98	0.009	0.999	0.665-1.148
BMI (kg/m^2)^	29.52±5.03	28.72±4.12	0.012	0.969	0.940-0.998
Charlson comorbidity index	2.3± 1.8	1.97± 1.69	0.008	0.915	0.776-1.078
Gender (n (%))			0.157	0.874	0.665-1.148
Male	281 (61.6)	175 (38.4)			
Female	334 (66.0)	172 (34.0)			

*p value of univariate analysis

Adjusted OR**: logistic regression analysis to predict discordance in the diagnosis of flat foot between Chippaux-Smirak index and Clarke´s angle adjusting for different variables

CI: Confidence interval

SD: Standard Deviation

## Discussion

The randomly studied sample had a high prevalence of excess weight and obesity that increased with age, consistent with population studies at both national and international level ^17^ ^,^ ^18^. In turn, and as would be expected, the comorbidity expressed by the Charlson score was higher in the eldest age group.

The prevalence of flat feet according to the podoscope was 19.0% in the left footprint (21.5% in women and 16.2% in men) and 18.9% in the right footprint (19.8% in women and 17.9% in men). In other population studies (Springfield, Massachusetts) the prevalence of flat feet was 19.0% (20.1% in women and 17.2% in men) ^19^. Another study carried out in the Boston area found a prevalence of 20% in women and 17% in men ^20^.

Some studies describe how podological pathologies increase with age ^21^, while others describe how flat foot decreases with age after adjusting for other covariables ^22^, and others indicate that neither age, gender or BMI are related to flat feet ^23^.

It is obvious that there is major variability, not only with regard to the characteristics of the sample studied but also the procedures used to diagnose flat feet and the age groups studied. In a Cochrane review in children, it was found that the variability is so great that some studies indicate prevalences of flat feet that vary between 0.6 -77.9% ^24^.

Flat foot has been described as becoming less prevalent in children with age ^25^ ^,^ ^26^, with some authors indicating a prevalence of 14% ^2^. Garcia-Rodríguez ^26^ reported a prevalence rate of 2.7% in 1,181 children between the ages of 2 and 13. Pfeiffer ^25^ reported that the prevalence of flexible flatfoot in children between the ages of 3 and 6 was 44.0%, although the prevalence of pathological flat feet was less than 1%.

If we use the Chippaux-Smirak index or Clarke's angle to diagnose flat feet, the prevalence values increase significantly. For both the Chippaux-Smirak index and Clarke's angle, the prevalence of flat feet is higher in the 65+ age group. 

This study highlights the limited concordance between Clarke's angle and the Chippaux-Smirak index in diagnosing pathologies of the foot arch. The same applies to both the left and right feet. Several authors have referred to the lack of concordance between the different procedures ^14^, noting that variations are found in footprint measurements collected using different techniques.

Despite the presence of variability between the procedures, there is also a high intra-rater reliability in different publications, using these indices ^14^ ^,^ ^27^.

We used the Chippaux-Smirak index as the benchmark in comparison with Clarke's angle, as the Chippaux-Smirak index takes three measurements of the footprint, while Clarke's angle only takes two measurement to diagnose pathologies of the foot arch. Some authors have even stated that the Chippaux-Smirak index has a better predictive capacity for diagnosing flat feet than Clarke's angle or the Staheli index ^5^. The variability found in part can be explained by the fact that these two measurements take different points of reference.

Other authors have even recommended using the Chippaux-Smirak index as a screening instrument for flat feet in preschool-age children ^6^ although there is no sound support for either continuing or establishing podiatry screenings for children ^7^. 

The Chippaux-Smirak index has been used as a standard assessment tool for determining whether preschool-age children suffered from flat foot ^8^.

This study reveals the limited sensitivity of Clarke's angle for diagnosing flat feet, using the Chippaux-Smirak index as a reference.

There is not only variability in the diagnosis, but also in the therapeutic management of flat feet; a Cochrane review concluded that there is no evidence from randomised controlled trials on the efficacy of foot orthoses for asymptomatic paediatric pes planus ^2^.

Digitalisation or electronic pedography procedures reduce variability in the measurements, but do not eliminate them completely ^14^, and so for this reason, identifying the variability of footprint measurement will aid us in the appropriate clinical foot posture assessment.

## Conclusions

This study highlights the variability found in observations for the diagnosis of flat feet and the limited concordance between Clarke's angle and the Chippaux-Smirak index for diagnosing pathologies of the foot arch. The findings are consistent in the 40-64 age group and in the group aged 65 and older. It will be necessary to reach consensus on and validate diagnostic procedures in order to reduce this clinical variability in diagnosing these patients. 
